# Human endothelial cells promote arsenic-transformed lung epithelial cells to induce tumor growth and angiogenesis through interleukin-8 induction

**DOI:** 10.18632/aging.203930

**Published:** 2022-03-03

**Authors:** Lei Zhao, Yi-Fang Wang, Jie Liu, Bing-Hua Jiang, Ling-Zhi Liu

**Affiliations:** 1 Department of Medical Oncology, Sidney Kimmel Cancer Center, Thomas Jefferson University, Philadelphia, PA 19107, USA; 2 Department of Pathology, Anatomy and Cell Biology, Thomas Jefferson University, Philadelphia, PA 19107, USA

**Keywords:** arsenic, lung cancer, angiogenesis, IL-8, paracrine

## Abstract

Arsenic exposure is associated with lung cancer. Angiogenesis is essential for tumor development. However, the role and mechanism of human vascular endothelial cells in tumor growth and angiogenesis induced by arsenic-transformed bronchial epithelial (As-T) cells remain to be elucidated. In this study, we found that endothelial cells significantly increased As-T cell-induced tumor growth compared to those induced by As-T cells alone. To understand the molecular mechanism, we found that endothelial cells co-cultured with As-T cells or cultured in conditioned medium (CM) prepared from As-T cells showed much higher cell migration, proliferation, and tube formation compared to those co-cultured with BEAS-2B (B2B) cells or cultured in CM from B2B. We identified that higher levels of intracellular interleukin 8 (IL-8) were secreted by As-T cells, which activated IL-8/IL-8R signaling to promote endothelial cells migration and tube formation. IL-8 silencing and knockout (KO) in As-T cells, or IL-8 neutralizing antibody dramatically suppressed endothelial cell proliferation, migration, tube formation *in vitro*, and tumor growth and angiogenesis *in vivo*, suggesting a key role of IL-8 in As-T cells to induce angiogenesis via a paracrine effect. Finally, blocking of IL-8 receptors C-X-C chemokine receptor type 1 (CXCR1) and CXCR2 with neutralizing antibodies and chemical inhibitors inhibited tube formation, indicating that IL-8Rs on endothelial cells are necessary for As-T cell-induced angiogenesis. Overall, this study reveals an important molecular mechanism of arsenic-induced carcinogenesis, and suggests a new option to prevent and treat arsenic-induced angiogenesis.

## INTRODUCTION

Arsenic is one of the most abundant and widely distributed semi-metallic elements in the crust of the earth. Humans are exposed to arsenic mainly through drinking inorganic arsenic-contaminated water, and inhalation and skin contact are also important ways [1, 2]. Mining is the predominant source of arsenic pollution, and the occupational exposure is mainly through industrial manufactures such as wood preservatives, mining activities, metal processing, and pesticides [2–4]. Arsenic exposure is a major public health concern and is associated with various diseases such as diabetes mellitus, hypertension, and cardiovascular diseases. Arsenic is also a well-documented carcinogen, and long-term exposure to arsenic is associated with lung, skin, urinary bladder, kidney, and liver cancers [5–7]. It has been reported that arsenic induces DNA damage by producing reactive oxygen and nitrogen species and changes gene expression via epigenetic mechanisms such as DNA methylation and histone modification [6, 8], which are closely related to arsenic-induced cancer. However, the mechanisms of arsenic-caused carcinogenesis still remain to be elucidated.

Angiogenesis, a process of generating new blood vessels via sprouting of pre-existing vasculature, is essential for tumor growth, invasion, and metastasis through transporting nutrients, oxygen, metabolites, and growth factors to the tumor. When tumors grow up to about 2 mm in diameter, they can no longer obtain enough oxygen and nutrients from surrounding environments simply by diffusion [9, 10]. Therefore, tumor angiogenesis is critical for further growth and progression of the tumor. Increased angiogenesis has been observed by arsenic exposure [11]. However, most of the current studies focus on the direct effect of arsenic on tumor angiogenesis or on the activity of endothelial cells by studying the transformed cells or vascular endothelial cells alone [12–14]. Notably, how arsenic-induced transformed bronchial epithelial (named As-T) cells trigger and maintain angiogenesis is poorly understood in arsenic-caused lung cancer.

The tumor microenvironment includes vasculature, cancer-associated fibroblasts (CAFs), extracellular matrix (ECM), and infiltrating immune cells, and their interaction with cancer cells has been considered to play an important role in cancer initiation, promotion, and progression [15, 16]. To trigger tumor angiogenesis, cancer cells secrete high levels of pro-angiogenic factors such as vascular endothelial growth factor (VEGF), platelet-derived growth factor (PDGF), and basic fibroblast growth factor (bFGF) into the tumor microenvironment [3]. These factors can activate quiescent endothelial cells of the surrounding blood vessels to promote the formation of new blood vessels. Extensive studies have demonstrated that the interactions between cancer cells, immune cells and vascular cells play key roles in promoting tumor growth and angiogenesis [15–18]. However, how As-T cells promote angiogenesis through modulating tumor microenvironments is still largely unknown.

Interleukin-8 (IL-8), a member of the chemokine family, plays an important role in tumor growth, angiogenesis, and metastasis [19]. Arsenic has been shown to induce IL-8 upregulation in kidney cells [20], arsenic-transformed urothelial cells [21], and arsenic-transformed human bronchial epithelial (HBE) cells [22]. Tumor microenvironmental IL-8 plays an important role in promoting tumor angiogenesis in lung cancer, colorectal cancer, and glioma [23] by directly enhancing endothelial cell proliferation, survival, and migration [24], but little information is known about how crosstalk between arsenic-transformed cells and vascular endothelial cells affects tumor growth and angiogenesis through IL-8. In the present study, we found that As-T cells and vascular endothelial cell interaction promoted tumor growth and angiogenesis. As-T cells generated more IL-8 to promote tumor angiogenesis through its paracrine effect; and IL-8 receptors, C-X-C chemokine receptor type 1 (CXCR1), and CXCR2, are required for As-T cells to induce endothelial cell proliferation, migration, and tube formation, thus enhancing tumor angiogenesis. This study sheds light on the important mechanism of microenvironmental crosstalk between arsenic-induced malignant transformed cells and vascular endothelial cells in mediating tumor angiogenesis and will be helpful to develop interventions targeting the IL-8 paracrine pathway to prevent and treat arsenic-induced lung cancer development in the future.

## RESULTS

### Human endothelial cells greatly promoted As-T cells to induce tumor growth

**Figure 1:**
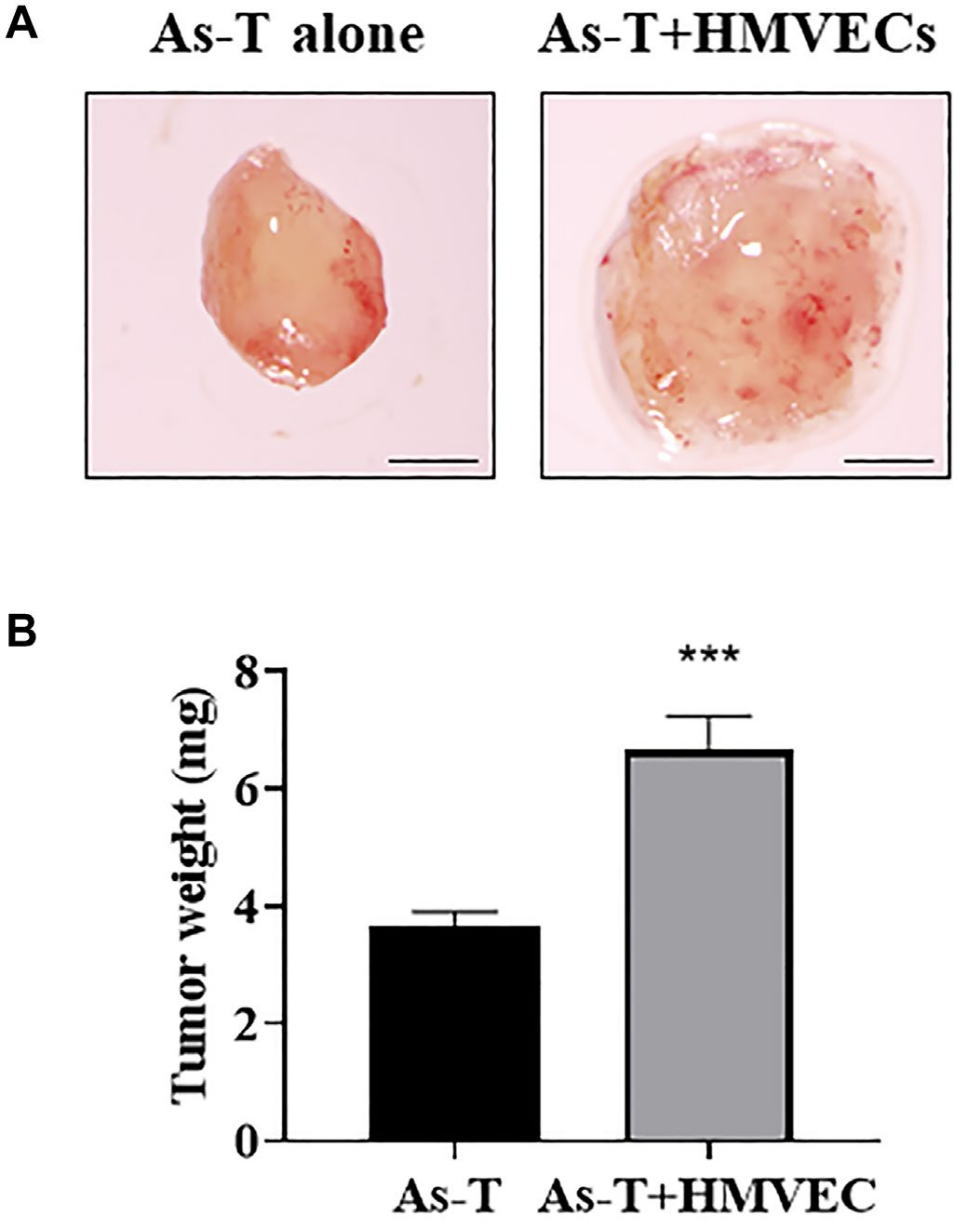
**Human endothelial cells greatly promoted As-T cells to induce tumor growth.** As-T cells and human dermal microvascular endothelial cells (HMVECs) were trypsinized, mixed in a 1:9 ratio with serum-free medium, and carried by the PLGA sponges for the implantation onto the CAM of 8 days old chicken embryos. As-T cells alone were used as a control. The tumors were harvested 12 days after the implantation. (**A**) Representative tumors. Scale, 1 mm. (**B**) The weight of plugs (*n* = 7 for each group). ^***^p < 0.001 compared with As-T alone control group.

Angiogenesis is essential for tumor growth, and activation of quiescent endothelial cells is the initial step of new blood vessel formation [25]. In order to study whether human vascular endothelial cells promote As-T cells to induce tumor growth, As-T cells were mixed with or without human dermal microvascular endothelial cells (HMVECs) in the presence of Matrigel, seeded into biodegradable sponges, and transplanted onto the chicken chorioallantoic membrane (CAM) to grow the tumor for 11 days. We found that the presence of HMVECs significantly increased tumor growth induced by As-T cells by more than 2-fold when compared to those tumors formed by As-T cells alone (Figure 1), indicating that endothelial signaling pathway(s) of HMVECs plays an important role in enhancing tumor growth.

### As-T cells increased cell migration and proliferation of human umbilical vein cells (HUVECs) and HMVECs

**Figure 2:**
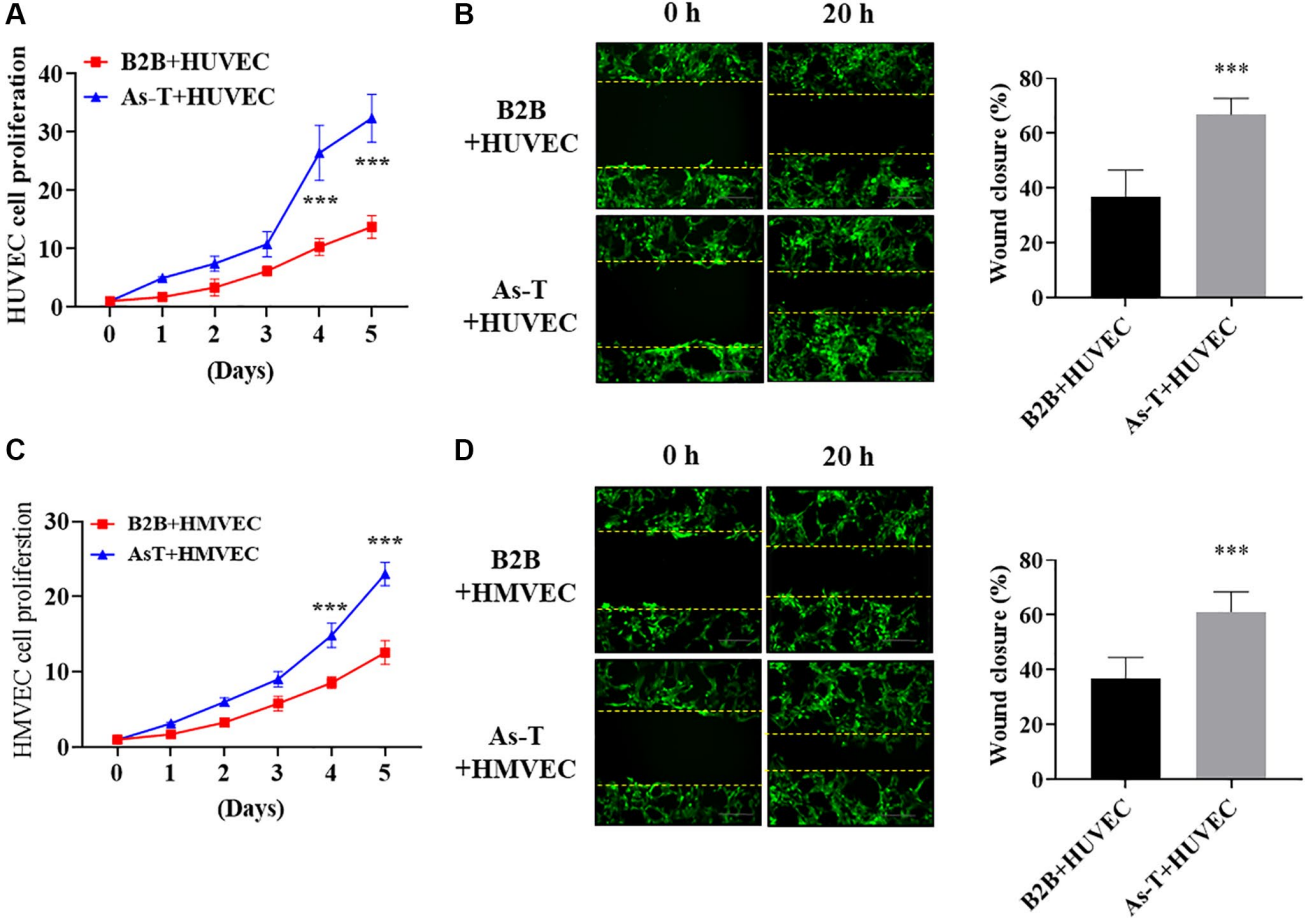
**As-T cells increased cell migration and proliferation of human umbilical vein cells (HUVECs) and HMVECs.** HUVECs or HMVECs were transduced by lentivirus expressing GFP and selected with puromycin. The GFP-positive HUVECs or HMVECs were then co-cultured with B2B or As-T cells in a 1:1 ratio. (**A**) The growth curve of HUVECs was plotted by counting the GFP-positive cells on indicated days. ^***^p < 0.001 compared to B2B+HUVEC group. (**B**) When reached about 100% confluence, the cells were starved overnight and a scratch wound was made with a 200 μL pipet tip. Cells were washed using PBS three times and 2 mL fresh basic EBM2 medium was added to the wells. The width of the wound was measured at 0 h and 20 h post scratch. Images were acquired at 10× magnification, bar = 100 μm. Left panel: representative images; right panel: quantification of wound healing closure from three experiments. ^***^p < 0.001 compared to B2B+HUVEC group. (**C**) The growth curve of HMVECs was plotted by counting the GFP-positive cells on indicated days. ^***^p < 0.001 compared to B2B+HMVEC group. (**D**) The wound healing assay for HMVEC cells was performed as described above. Images were acquired at 10× magnification, bar = 100 μm. ^***^p < 0.001 compared to B2B+HMVEC group.

To investigate the molecular mechanism of vascular endothelial cells to induce tumor growth, we co-cultured immortalized human bronchial epithelial cells BEAS-2B (B2B) or As-T cells with HUVECs expressing green fluorescent protein (eGFP). We found that co-culture of HUVECs with As-T cells increased HUVECs proliferation on Day 4 and Day 5 by 2-fold when compared to that of co-cultured HUVECs with B2B cells (Figure 2A). Wound healing assay results showed that co-culture with As-T cells also markedly increased the migration ability of HUVECs by about 40% more wound closure (Figure 2B). Similarly, co-culture of HMVECs with As-T cells significantly increased the cell proliferation (Figure 2C) and migration ability (Figure 2D) of HMVECs compared to co-culture with B2B cells. The results suggest that transformed cells may secret more factors to activate vascular endothelial cells and increase endothelial cell migration and proliferation that are parts of the angiogenesis process.

### Vascular endothelial cells cultured in conditioned medium (CM) from As-T cells showed higher cell viability, more cell migration, and better tube formation

**Figure 3:**
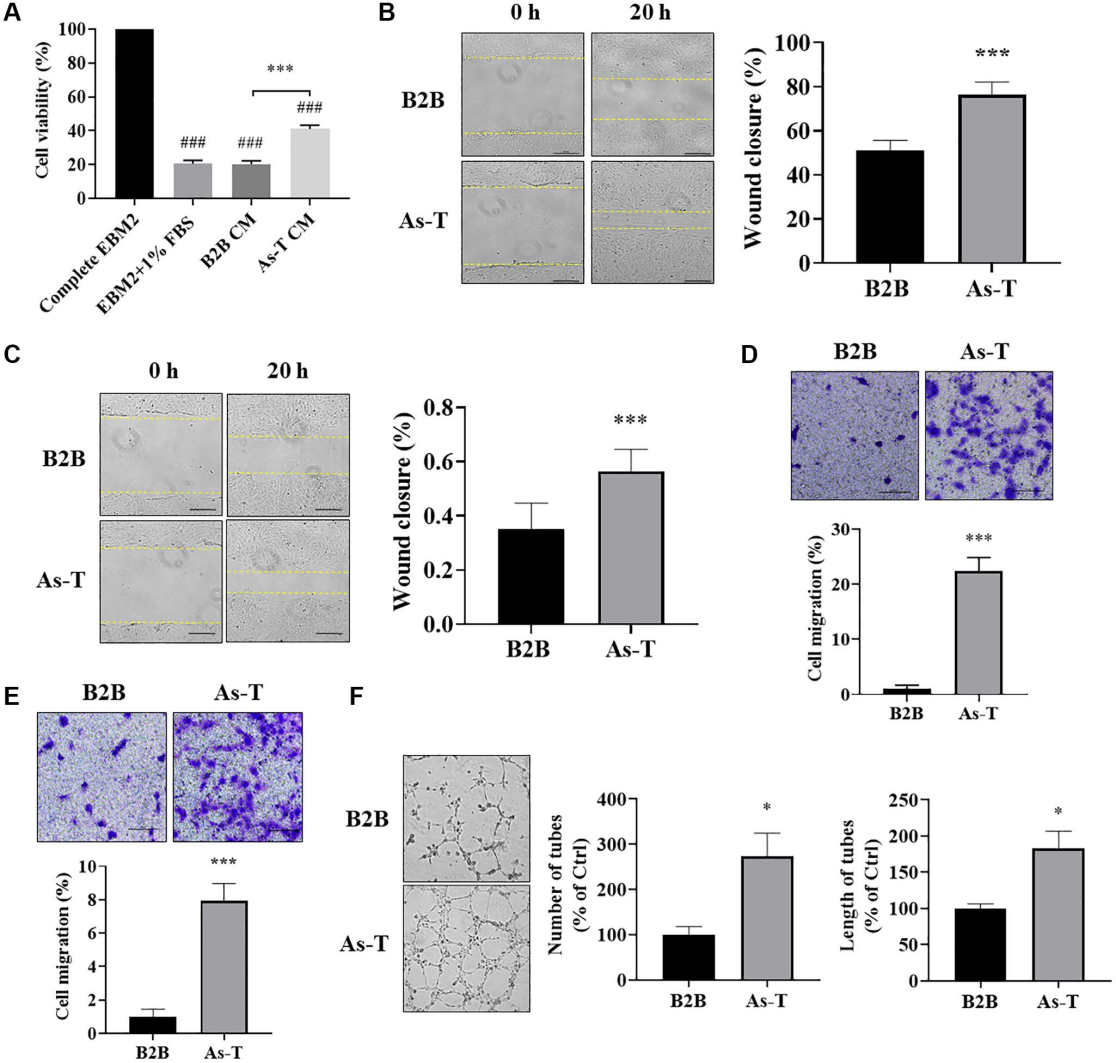
**Vascular endothelial cells cultured in conditioned medium (CM) from As-T cells showed higher cell viability, more cell migration, and better tube formation.** (**A**) HUVECs were incubated with the CM from B2B or As-T cells for 4 days. Cell proliferation of HUVECs was determined by counting the number of trypan blue negative cells. ^###^*p* < 0.001 compared to complete EBM2 medium group; ^***^p < 0.001 compared to B2B CM group. (**B**) When reached about 100% confluence, HUVECs were starved overnight, and a scratch wound was made as above. Superannuant was removed, and the CM from B2B or As-T cells was added to the wells. The widths of the wounds were measured at 0 h and 20 h post scratch. Images were taken using an inverted microscope at 10× magnification, bar = 100 μm. Left panel: representative images; right panel: quantification of the wound healing assay. ^***^p < 0.001 compared to CM from the B2B group. (**C**) Wound healing assay for the HMVECs was performed as described above. Bar = 100 μm, ^***^p < 0.001. (**D**) A transwell assay was conducted to evaluate the effects of CM on HUVEC migratory ability. HUVECs were plated on the top chamber in 200 μL basic EBM2 medium; the lower chamber was filled with 600 μL CM from B2B or As-T cells. The cells that pass through the network were stained with crystal violet and counted under a microscope at 24 h, and the images were taken using a microscope at 10× magnification, bar = 100 μm. Upper panel: representative images; lower panel: quantification of the migrated cells. ^***^p < 0.001 compared to CM from B2B cells. (**E**) Migration assay for the HMVECs was performed as described above. Bar = 100 μm, ^***^p < 0.001. (**F**) A tube formation assay was carried out to evaluate the effects of CM on the tube forming ability of HUVECs. HUVECs were starved with basic EBM2 medium overnight and resuspended in 100 μL diluted CM (CM: basic EBM2 = 1:1). The cell suspension was then added to a 96 well-plate containing 100 μL solidified growth factor-reduced Matrigel. The tubular structures were imaged at about 6–12 h under a microscope at 4× magnification. Left panel: representative images of the tubular structures. Middle panel: quantification of the number of the tubular structures. Right panel: quantification of the length of the tubular structures. ^*^p < 0.05 compared to CM from B2B group.

Secretory factors, including growth factors and cytokines, play important roles in the growth and functions of vascular endothelial cells and are important mediators in the tumor microenvironment for the crosstalk between different types of cells. In order to compare the effect of CM from normal and transformed cells on vascular endothelial cellular function, we collected the CM from B2B and As-T cells cultured in a basic EBM2 medium with 1% FBS to reduce the growth factors. The HUVECs were cultured in the CM for four days, and the cell viability was determined by counting the number of living cells. We found that compared to EBM2 with 1% FBS and CM from B2B cells, the CM from As-T cells significantly increased the growth of HUVECs (Figure 3A). Furthermore, compared to the CM from B2B, the CM from As-T cells dramatically promoted the migration ability of both HUVECs and HMVECs, showing more wound closure in the wound healing assay and more transwelled cells in the transwell migration assay (Figure 3B–3E). Consistent with these results, we also found that As-T cells growing in EBM2 with 1% FBS in the lower chamber attracted more HUVECs to pass through the mesh (Supplementary Figure 1). The tube formation assay showed that HUVECs cultured in CM from As-T cells formed better tubular structures with more number and length of tubes, further confirming that the CM of As-T cells may contain higher levels of angiogenic factors to promote angiogenesis than that of B2B cells (Figure 3F). Overall, the results strongly indicate that secretory factor(s) released by As-T cells mediates the communication between As-T cells and vascular endothelial cells to promote vascular endothelial cell proliferation and angiogenesis.

### As-T cells released higher level of IL-8, which activated the IL-8 pathway in endothelial cells via the paracrine effect

**Figure 4:**
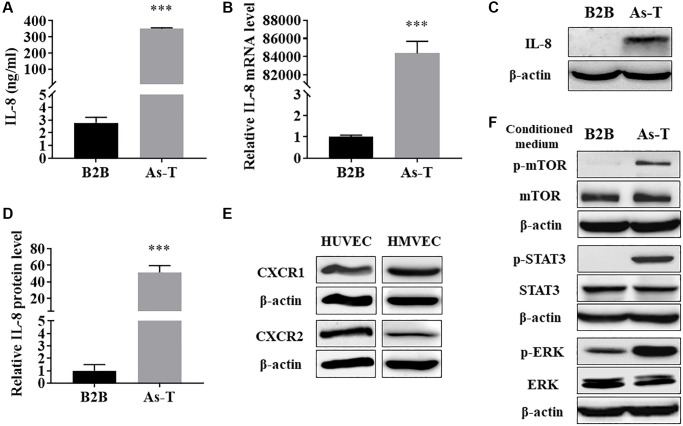
**As-T cells released higher level of IL-8, which activated the IL-8 pathway in endothelial cells via the paracrine effect.** (**A**) The protein levels of IL-8 in CM from B2B and As-T cells were measured using ELISA assay. ^***^p < 0.001 compared to B2B cells. (**B**) The mRNA level of IL-8 was measured using qRT-PCR. ^***^p < 0.001 compared to B2B cells. (**C**) The protein level of IL-8 in B2B and As-T cells was determined using immunoblotting assay. (**D**) Quantification of the immunoblotting results using Image Lab software. ^***^p < 0.001 compared to B2B. (**E**) The protein expression of IL-8 receptors, CXCR1 and CXCR2, in HUVECs (left) and HMVECs (right) was determined using immunoblotting assay. (**F**) HUVECs were starved overnight and then treated with CM from B2B or As-T cells for 6 h. The expression of IL-8/IL-8R signaling pathway molecules, p-mTOR, mTOR, p-STAT3, STAT3, p-ERK, ERK, and β-actin was analyzed using immunoblotting assay.

To identify angiogenic inducer(s) in the CM of As-T cells compared to parental B2B cells, we compared the differential expression levels of factors in CM prepared from As-T and B2B cells using Angiogenesis Protein Arrays (RayBiotech, Peachtree Corners, GA, USA). We found that IL-8 was one of the most significantly upregulated angiogenic factors in the CM of As-T cells compared with CM of B2B cells (data not shown). IL-8 has been demonstrated to play an important role in tumor angiogenesis by directly promoting endothelial cell survival and proliferation [24]. To verify the result of IL-8 expression level in CM of As-T by Angiogenesis Protein Array, we demonstrated that protein level of IL-8 in the CM from As-T cells was dramatically increased to more than 100-fold compared to that from parental B2B cells using ELISA assay (Figure 4A). Accordingly, the expression of IL-8 in As-T cells was significantly increased at both mRNA (Figure 4B) and protein (Figure 4C and 4D) levels. Together, these results suggest that As-T cells express a higher level of IL-8 and thereby secrete more IL-8 into the supernatant. Activation of the IL-8/IL8R signaling pathway plays an important role in endothelial cell function and angiogenesis. To investigate whether the secreted IL-8 by As-T cells activates the IL-8/IL8R signaling pathway in vascular endothelial cells via a paracrine mechanism, we found that HUVECs and HMVECs express both CXCR1 (IL-8RA) and CXCR2 (IL-8RB, Figure 4E). Treatment with CM of As-T led to the activation of several downstream targets of the IL-8/IL8R signaling pathway, including p-mTOR, p-STAT3, and p-ERK (Figure 4F) in HUVEC cells. These results demonstrate that compared to parental B2B cells, As-T cells produce much higher level of IL-8 and secret it into supernatant to activate IL-8/IL8R signaling pathway in vascular endothelial cells, thus promoting angiogenesis through the paracrine effect.

### IL-8 generated by As-T cells was required for angiogenesis *in vitro* and *in vivo*

**Figure 5:**
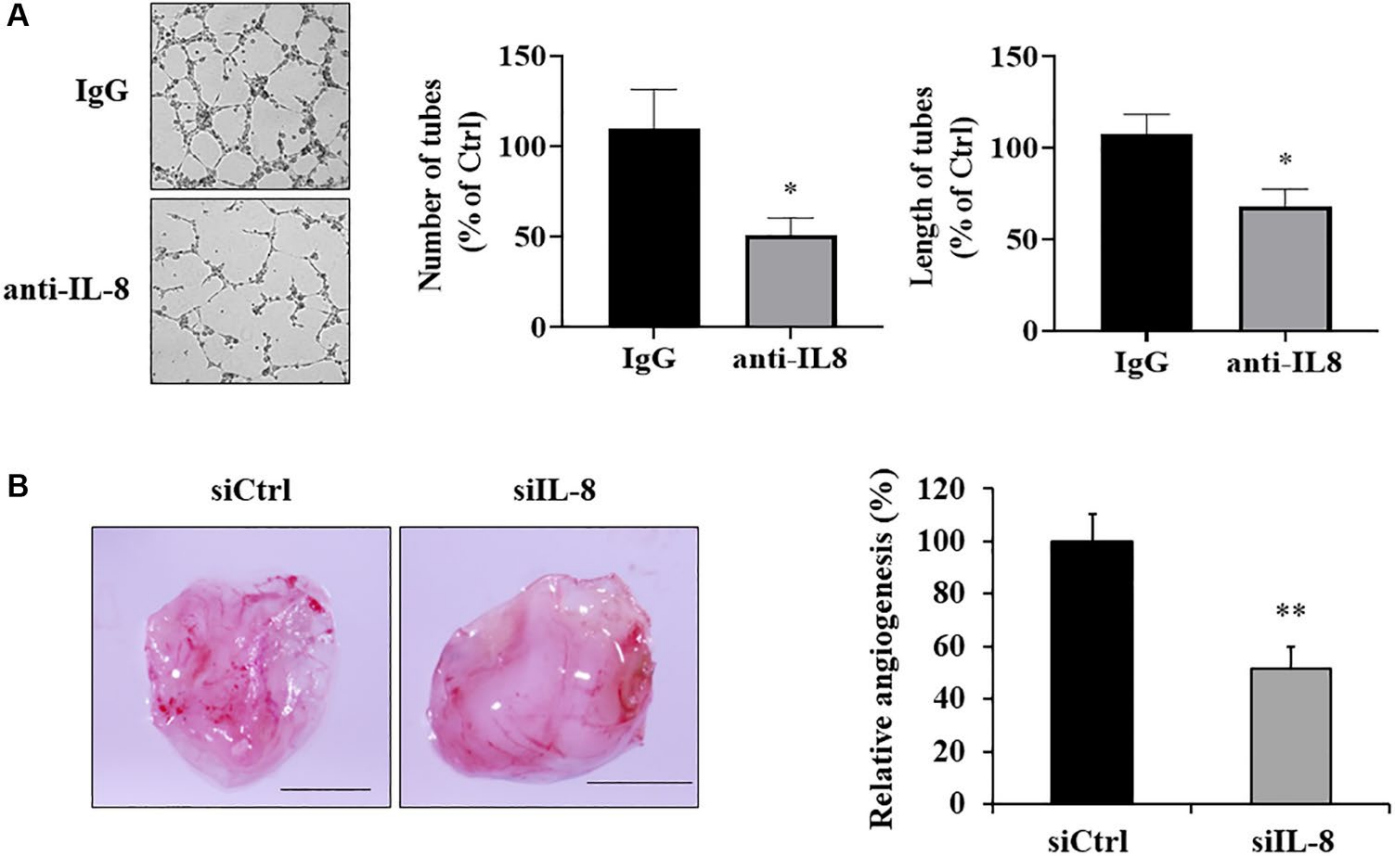
**IL-8 generated by As-T cells was required for angiogenesis *in vitro* and *in vivo.*** (**A**) IgG or anti-IL-8 neutralizing antibody was added to the CM of As-T cells and incubated for 30 min, then the CM was mixed with starved HUVECs in basic EBM2 in a 1:1 ratio to perform the tube formation assay as described above. The images of the tubular structures were captured 6–12 h later using an inverted microscope at 4× magnification. Left panel: representative images of the tubular structures. Middle panel: quantification of the number of the tubular structures. Right panel: quantification of the length of the tubular structures. ^*^p < 0.05 compared to IgG control. (**B**) IL-8 was knocked down in As-T cells by transfection of siRNA against IL-8, and angiogenesis assay was performed to evaluate the effects of IL-8 silencing on angiogenesis using CAM model. Left panel: representative plugs from siRNA scrambled control (siCtrl) and siIL-8 groups. Scale is 2 mm. Right panel: the number of blood vessel branches was counted from eight replicates and normalized to that of the control group. ^**^p < 0.01 compared to the siCtrl group.

Next, in order to determine whether IL-8 generated by As-T cells is necessary for promoting angiogenesis, we added an anti-IL-8 neutralizing antibody into CM of As-T cells that interfered with the binding of IL-8 to its receptors on vascular endothelial cells. Tube formation assay results showed that compared to IgG treatment, CM from As-T cells treated with IL-8 neutralizing antibody impeded the tube formation capacity of HUVECs with 40% to 50% of inhibition (Figure 5A), indicating that secreted IL-8 by As-T cells contributes to angiogenesis. To further investigate whether IL-8 generation in As-T cells is required for As-T cell-induced angiogenesis *in vivo*, we used siRNA against IL-8 to knock down IL-8 expression in As-T cells and performed angiogenesis assay using CAM model. The results showed that compared to scrambled control siRNA (siCtrl), IL-8 silencing in As-T cells significantly inhibited angiogenesis by 50% with fewer branches of microvessels (Figure 5B), indicating that IL-8 is indispensable in the As-T cells to induce angiogenesis.

### IL-8 knockout in As-T cells impeded vascular endothelial cell proliferation, migration, and tube formation

**Figure 6:**
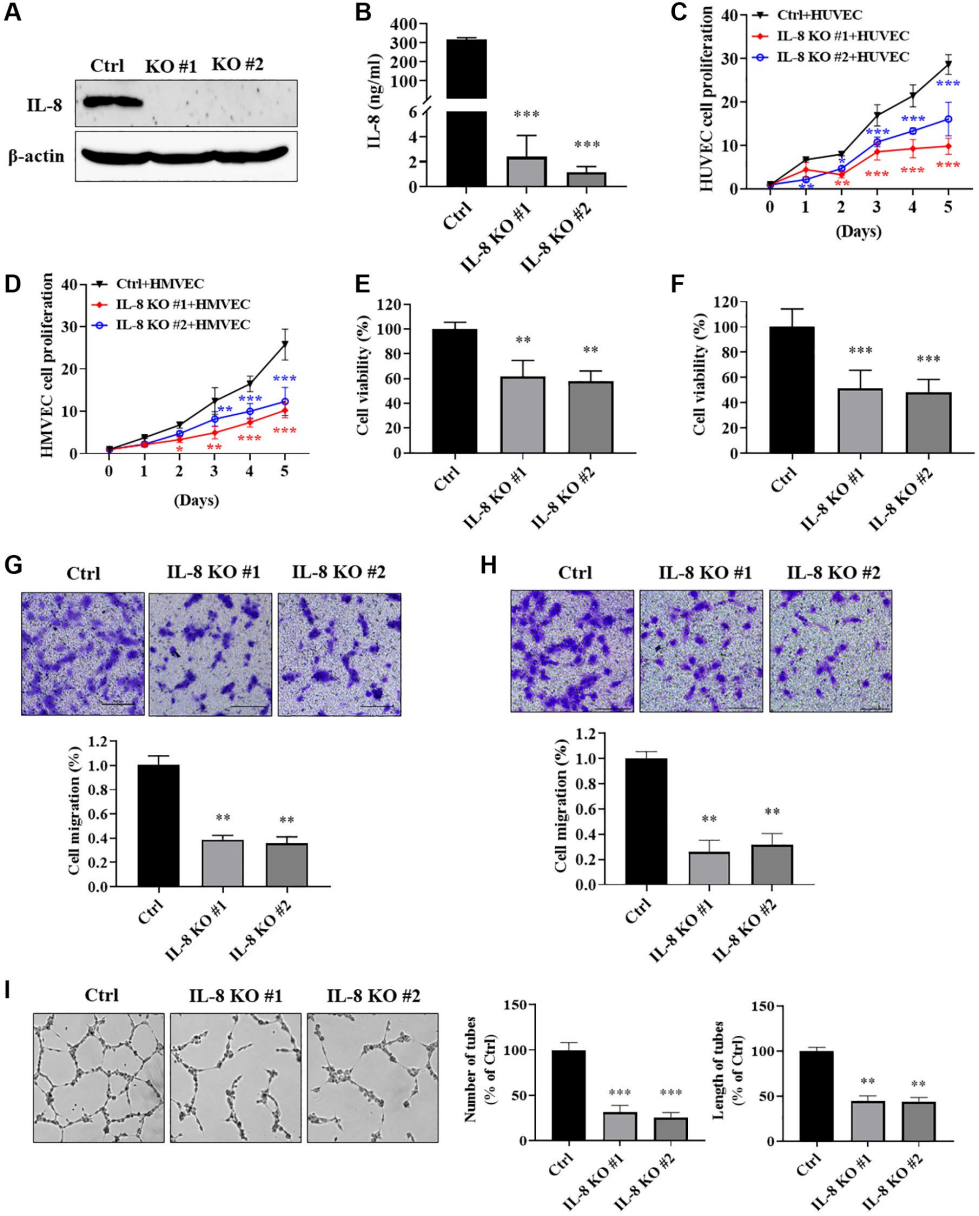
**IL-8 knockout in As-T cells impeded vascular endothelial cell proliferation, migration, and tube formation.** We generated As-T IL-8 knockout (KO) and control (Ctrl) stable cells using the CRISPR/Cas9 technique. (**A**) Complete depletion of IL-8 in As-T cells was confirmed using immunoblotting assay. (**B**) The protein levels of IL-8 in the CM from As-T Ctrl and two IL-8 KO stable cell lines were measured using ELISA assay. (**C** and **D**) GFP-positive HUVECs (**C**) or HMVECs (**D**) and As-T Ctrl or IL-8 KO cells were mixed in a 1:1 ratio and seeded into 6-well plates. The number of GFP-positive cells was counted on indicated days. ^**^ and ^***^p < 0.01 and *p* < 0.001, respectively, compared to the As-T Ctrl group. (**E** and **F**) HUVECs (**E**) or HMVECs (**F**) were incubated with the CM from As-T Ctrl and IL-8 KO cells for 4 days. The cell viability was determined by counting the number of trypan blue negative cells. ^**^ and ^***^p < 0.01 and *p* < 0.001, respectively, compared to the CM from As-T Ctrl group. (**G** and **H**) Transwell migration assays were performed to evaluate the effects of IL-8 KO on HUVECs (**G**) or HMVECs (**H**) migratory ability. HUVECs or HMVECs were plated on the top chamber in 200 μL basic EBM2 medium; the lower chamber was filled with 600 μL CM from As-T Ctrl or IL-8 KO cells. The cells that passed through the membrane were stained with crystal violet and counted under the microscope after 24 h. The images were taken at 10× magnification. Upper panel: representative images; bottom panel: quantification of migrated cells. ^**^p < 0.01 compared to the wild type As-T Ctrl group. (**I**) A tube formation assay was performed using the CM from control and IL-8-null cells as described above. The images of the tubular structures were taken using an inverted microscope at 4× magnification. Left panel: representative images of the tubular structures. Middle panel: quantification of the number of the tubular structures. Right panel: quantification of the length of the tubular structures. ^**^ and ^***^p < 0.01 and *p* < 0.001, respectively, compared to CM from wild type As-T Ctrl cells.

To investigate how cellular IL-8 in As-T cells regulates the biological function of vascular endothelial cells, we knocked out (KO) IL-8 expression in As-T cells using the CRISPR/Cas9 technique and evaluated its effect on endothelial cell proliferation and migration. The complete depletion of IL-8 was confirmed by immunoblotting assay (Figure 6A). IL-8 in the CM from the two IL-8 KO cell lines was almost abolished compared to the As-T Ctrl cells using ELISA assay (Figure 6B). Co-culture of HUVECs with IL-8-null As-T cells resulted in a significantly decreased cell proliferation rate of HUVEC compared to the co-culture with wild type As-T cells (Figure 6C). Similar results were observed in HMVECs co-cultured with IL-8-null As-T cells (Figure 6D). CM from IL-8 KO cells dramatically reduced the viability of HUVECs (Figure 6E) and HMVECs (Figure 6F) to 50% to 60% compared to that from control cells. We then performed a transwell assay to evaluate the effects of secreted IL-8 on vascular endothelial cell migratory ability. As we expected, significantly fewer HUVECs (Figure 6G) and HMVECs (Figure 6H) passed through the mesh when the lower chamber was filled with CM from IL-8-null As-T cells. The tube formation ability of HUVECs was dramatically compromised when the cells were cultured in the CM from IL-8-depleted cells, as the number and length of the tubular structures were attenuated to 20-40% compared to control (Figure 6I). These results suggest that IL-8 in As-T cells is required to secrete to the supernatant to promote vascular endothelial cell growth, migration, and tube formation under the co-culture condition.

### IL-8 knockout in As-T cells attenuated tumor growth and angiogenesis using HMVEC/As-T co-implantation animal model

**Figure 7:**
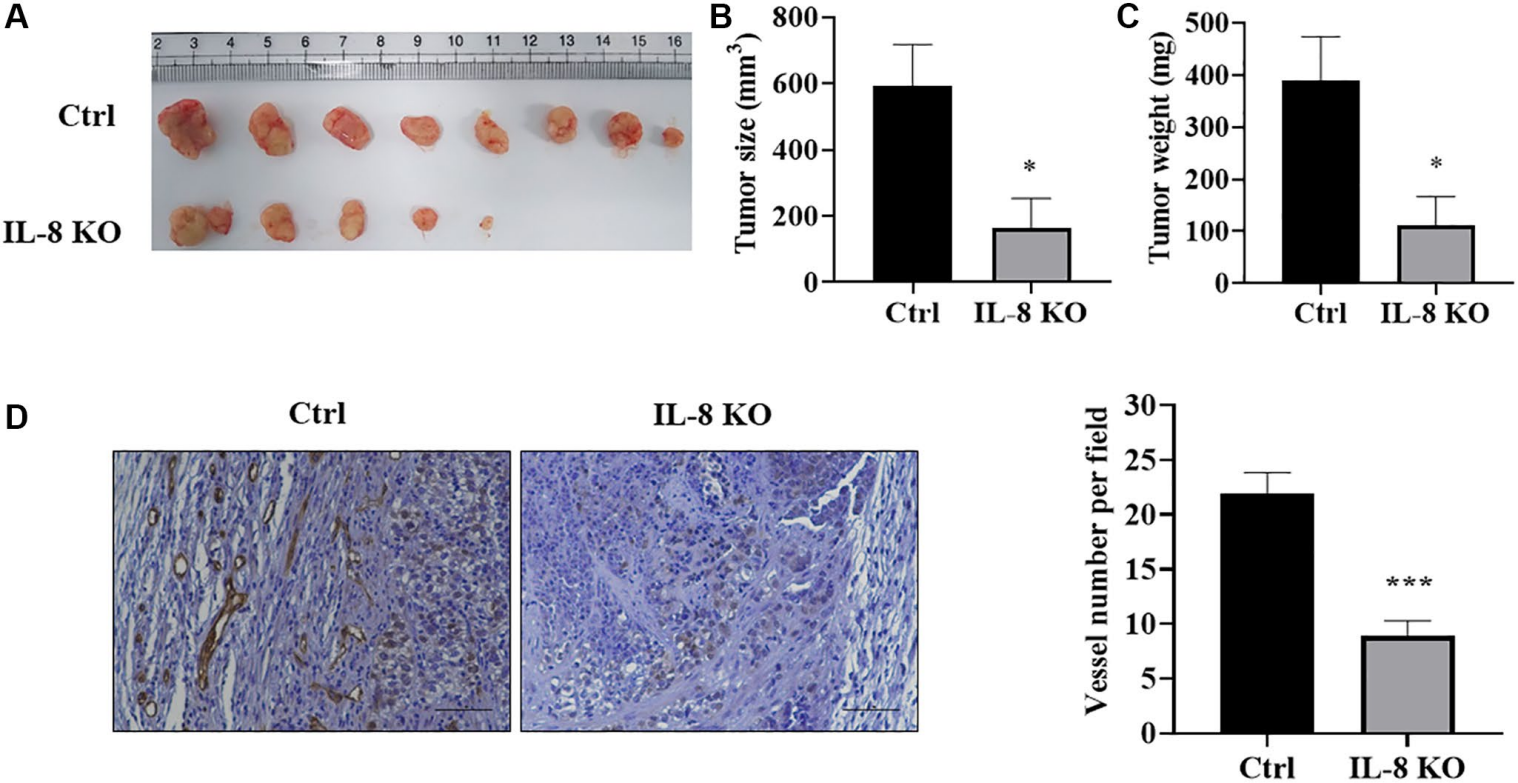
**IL-8 knockout in As-T cells attenuated tumor growth and angiogenesis using HMVEC/As-T co-implantation animal model.** IL-8 was knocked out in As-T cells using the CRISPR/Cas9 technique. The HMVEC cells (0.9 × 10^6^) were mixed with wild type Ctrl or IL-8 KO As-T cells (0.1 × 10^6^) in basic EBM2 medium and then mixed with 1:1 (v/v) growth factor-reduced Matrigel. The cell suspension was subcutaneously injected into flanks of nude mice at 6 weeks of age. The tumors were harvested 6 weeks after the implantation. (**A**) Representative tumors were shown. (**B**) The size of tumors. ^*^p < 0.05 compared to the As-T Ctrl+HMVECs group. (**C**) The weight of the tumors. ^*^p < 0.05 compared to the As-T Ctrl+HMVECs group. (**D**) IHC staining of CD31 in tumors. Left panel: representative images of IHC staining. Scale, 50 μm. Right panel: quantification of the microvessel structures in the IHC images. ^***^p < 0.001 compared to the Ctrl group.

In order to investigate whether IL-8 knockout in As-T cells affects tumor growth and angiogenesis by interfering with the crosstalk between As-T cells and vascular endothelial cells, we performed a tumor growth assay using HMVEC/As-T co-implantation animal model. The HMVECs and wild type control or IL-8-null As-T cells were mixed with Matrigel, then injected into both flanks of nude mice. The results showed that after six weeks, co-implantation of HMVECs with IL-8-null As-T cells resulted in less tumor growth and showed smaller size and less weight of xenografts when compared to co-implantation with wild type As-T cells (Figure 7A–7C). We also test microvessels by immunohistochemical staining of CD31. IL-8 knockout in As-T cells dramatically inhibited angiogenesis with about 60% decrease of CD31 positive microvessels (Figure 7D). Consistent with these results, when compared to co-implantation with As-T mock control cells, HMVECs co-implanted with As-T-siIL-8 cells significantly suppressed tumor growth using chimeric tumor model as shown in Figure 1 (Supplementary Figure 2). The *in vivo* study demonstrates that IL-8 in As-T cells is important for tumor growth and angiogenesis by interacting with vascular endothelial cells and maintaining the tumor microenvironment.

### IL-8 receptors in vascular endothelial cells were required for angiogenesis

**Figure 8:**
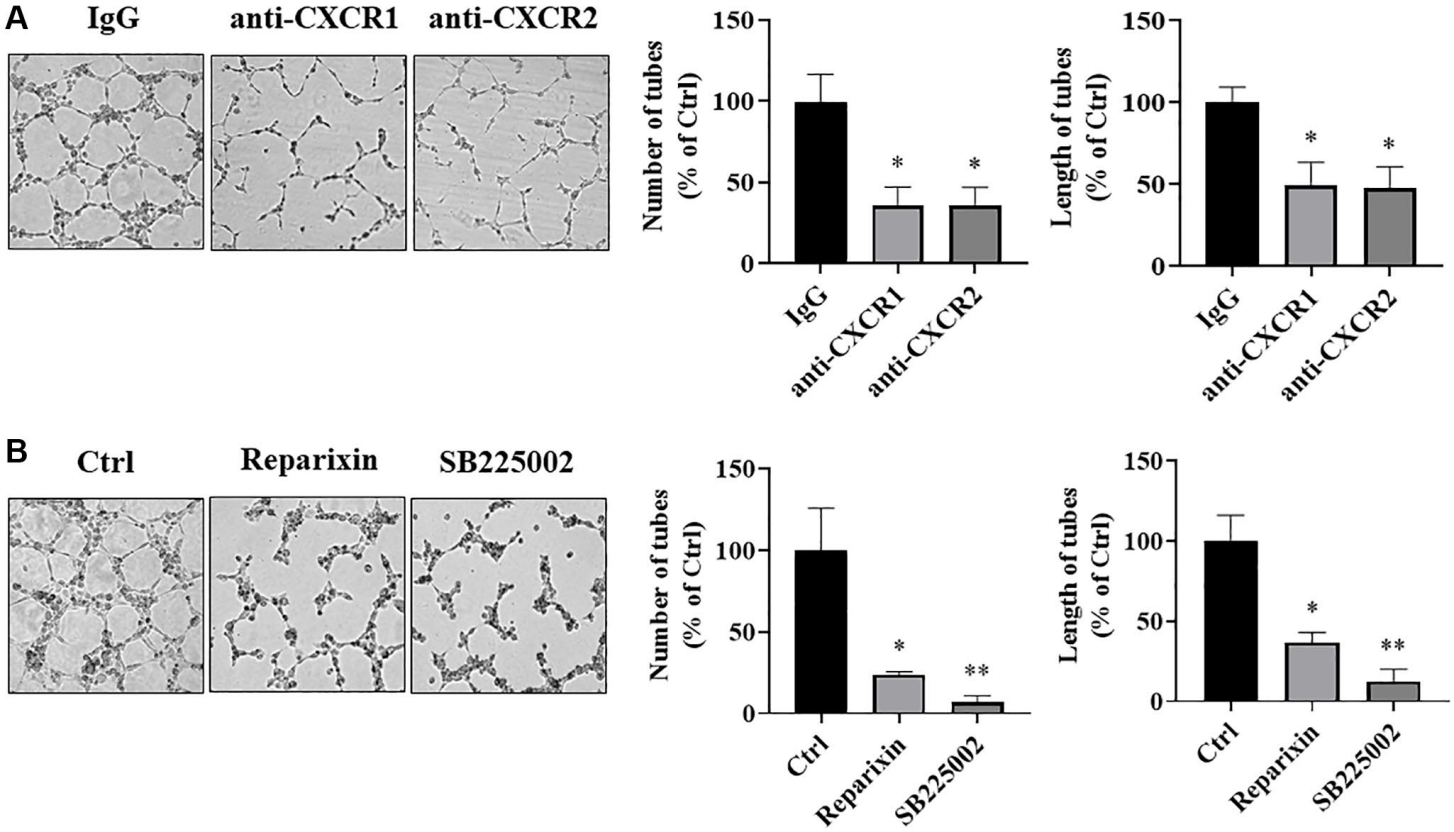
**IL-8 receptors in vascular endothelial cells were required for angiogenesis**. (**A**) HUVECs were starved overnight and resuspended in basic EBM2 medium supplemented with IgG or anti-CXCR1 or anti-CXCR2 neutralizing antibodies for 30 min. Then the cells were mixed with CM from As-T cells in a 1:1 ratio and plated in 96 well plates pre-coated with solidified growth factor-reduced Matrigel for tube formation assay. The tubular structures were imaged at 6–12 h at 4× magnification. Left panel: representative images of the tubular structure. Middle panel: the number of tubular structures was measured. Right panel: the length of the tubular structures was measured. ^*^p < 0.05 compared to the IgG control group. (**B**) HUVECs were starved overnight. The cells were then treated with DMSO, Reparixin (CXCR1 inhibitor), or SB225002 (CXCR2 inhibitor) for 2 h. The tube formation assay was performed as above. The tubular structures were imaged at 6–12 h using a microscope at 4× magnification. Left panel: the representative images of the tubular structure. Middle panel: quantification of the number of the tubular structures. Right panel: quantification of the length of the tubular structures. ^*^ and ^**^p < 0.05 and *p* < 0.01, respectively, compared to the DMSO treatment control group.

To further determine whether IL-8 secreted from transformed cells stimulates angiogenesis through a paracrine mechanism, we blocked two IL-8 receptors in HUVECs using both the neutralizing antibodies and chemical inhibitors. Tube formation assay showed that the number and length of the tubular structures formed by HUVECs were significantly inhibited by more than 50% when IL-8 receptors CXCR1 and CXCR2 were blocked by corresponding neutralizing antibodies (Figure 8A). Consistently, the formation of tubular structures was also attenuated when CXCR1 and CXCR2 on HUVECs were blocked by their chemical inhibitors, Reparixin and SB225002, respectively, and SB225002 exhibited a stronger inhibitory effect than Reparixin (Figure 8B). These results indicate that secreted IL-8 induces angiogenesis through binding to vascular endothelial CXCR1 and CXCR2.

## DISCUSSION

Among all the tumor microenvironmental cells, vascular endothelial cells are important for tumor angiogenesis, which is required for diffusion exchange of nutrients and metabolites to maintain tumor growth and promote cancer development and treatment resistance. It has been reported that the crosstalk between tumor cells and surrounding endothelial cells promotes angiogenesis and tumor growth in head and neck cancer, breast cancer, and liver cancer [26]. Arsenic is an important environmental carcinogen that causes several types of cancer. Previous studies including ours have demonstrated that long-term exposure to a low dose of arsenic leads to malignant transformation of human normal bronchial epithelial cells, and these arsenic transformed cells possess cancerous characteristics [14, 27–29]. Wang et al. have shown that arsenic-transformed human bronchial epithelial cells (HBECs) undergo epithelial-to- mesenchymal transition (EMT), which promotes angiogenesis via activating the β-catenin/VEGF pathway [14]. We and other researchers have shown that arsenic exposure promotes angiogenesis and tumor growth [30–32]. However, how the arsenic-transformed cells regulate angiogenesis through communication with vascular endothelial cells is largely unknown. In this study, we found that crosstalk between As-T cells and human dermal vascular endothelial cells dramatically promoted tumor growth compared to As-T cells alone, confirming the important effect of vascular endothelial cells in tumor microenvironment on tumor growth.

Many angiogenic factors, including VEGF, bFGF, TGF-α, TGF-β, TNF-α, platelet-derived endothelial growth factor (PDEGF), granulocyte colony-stimulating factor (GM-SF), placental growth factor, IL-8, and epidermal growth factor, promote the process of angiogenesis [33], and these angiogenic activators play important roles in the growth and spread of tumor cells [34]. In order to determine the most important angiogenic factor in mediating tumor angiogenesis through As-T and vascular endothelial cell interaction, we used Angiogenesis Protein Arrays to analyze the differential protein profile in conditioned media from As-T and parental B2B cells, and found that IL-8 was one of the most significantly upregulated angiogenic factors. IL-8 is a pro-inflammatory factor that belongs to the CXC subfamily of chemokines and functions as an important regulatory factor of the tumor microenvironment [19]. IL-8 secretion is induced by various environmental stress such as hypoxia, chemotherapeutic reagents, and heavy metals [35]. It is produced by a variety of cancer cells, leukocytes, and fibroblasts [35] to promote tumor cell proliferation, invasion, and metastasis [36, 37]. Arsenic has been shown to induce upregulation of IL-8 in kidney cells [20], arsenic-transformed urothelial cells [21], and arsenic-transformed HBECs [22]. Tumor cell-derived IL-8 can stimulate cancer cell proliferation and migration via the autocrine mechanism; and promote the growth and angiogenic ability of endothelial cells and the recruitment of neutrophils to the tumor site via the paracrine mechanism [38, 39]. To investigate the role and mechanism of IL-8 in As-T cells on mediating vascular endothelial functions and angiogenesis by crosstalk, we found that interaction between As-T and vascular endothelial cells HUVECs and HMVECs promoted vascular endothelial cell proliferation and migration. In consistent with previous studies showing the angiogenic role of IL-8 in the endothelial cell [24, 40], we found that compared to parental normal bronchial epithelial cells B2B, malignant As-T cells produced a very high level of IL-8 to secrete into the supernatant, which activated IL-8/IL-8R signaling in vascular endothelial cells through a paracrine mechanism to enhance cell viability, migration, and tube formation. Furthermore, IL-8 neutralizing antibody or siRNA against IL-8 in As-T cells dramatically inhibited angiogenesis *in vitro* and *in vivo*. IL-8 KO in As-T cells further abolished the capacities of cell viability, proliferation, migration, and tube formation of vascular endothelial cells via the paracrine effect, and markedly suppressed tumor growth and tumor angiogenesis using HMVEC/As-T co-implantation animal model. These results are consistent with previous results in colon cancer, glioblastoma, and ovarian cancer, in which IL-8 has been demonstrated to promote angiogenesis and tumor growth both *in vitro* and *in vivo* [41]. As far as we know, this is the first report to reveal that secreted IL-8 by As-T cells promotes tumor growth and angiogenesis through crosstalk with vascular endothelial cells via its paracrine mechanism.

IL-8 exerts its biological effects through binding to two types of cell-surface G protein-coupled receptors, CXCR1 and CXCR2 [42]. Upon IL-8 binding to its receptors, it activates the IL-8/IL-8R signaling pathway to induce the activation of the mitogen-activated protein kinase (MAPK)/ERK1/2 [43, 44], STAT3 [45, 46], and PI3K/AKT/mTOR signaling pathways [47]. These signaling pathways play important roles in cell proliferation, apoptosis, migration, invasion, and angiogenesis. In this study, we found that HUVECs and HMVECs expressed both CXCR1 and CXCR2, which is consistent with the previous study, showing that HUVEC and HMEC cells express CXCR1 and CXCR2 protein [24]. When we cultured HUVECs in CM from As-T cells, HUVECs showed increased phosphorylation of mTOR, STAT3, and ERK1/2, indicating upregulated IL-8 by As-T cells can effectively activate the IL-8/CXCR1/2 signaling pathway in vascular endothelial cells. Next, in order to study whether endothelial CXCR1 and CXCR2 are necessary for As-T cells-induced angiogenesis through the paracrine effect, blocking the binding of IL-8 to its receptor using neutralizing antibodies and chemical inhibitors showed a significant inhibitory effect on tube formation, suggest that IL-8 in As-T cells induces cellular function of vascular endothelial cells to promote angiogenesis through binding to the IL-8 receptors.

In summary, we found that arsenic transformed cells produced and secreted very high level of IL-8 to induce tumor growth and angiogenesis through As-T and vascular endothelial cell communication. IL-8 is one of the key angiogenic factors to promote tumor angiogenesis via the paracrine mechanism in As-T cells. The results from this study will extend our understanding of the mechanisms of lung cancer caused by long-term arsenic exposure and will provide potential targets for anti-angiogenesis therapies and cancer prevention by modulating tumor microenvironment in metal-induced carcinogenesis in the future.

## MATERIALS AND METHODS

### Cell culture and reagents

B2B cells were obtained from American Type Culture Collection (ATCC, Manassas, VA, USA). B2B cells were transformed into As-T cells by exposure to 1 μM sodium arsenic for 6 months as we previously described, which act like lung cancer cells and can form tumors in nude mice [29]. Passage-matched B2B cells were used as parental control cells. B2B and As-T cells were cultured in DMEM (Dulbecco’s modified Eagle’s medium; Invitrogen, Carlsbad, CA, USA) supplemented with 10% fetal bovine serum (FBS) and 50 U/ml Penicillin-Streptomycin. Human umbilical vein endothelial cells (HUVEC) and human dermal microvascular endothelial cells (HMVEC) were obtained from Lonza (Lonza, Hayward, CA, USA) and cultured in endothelial basal medium-2 (EBM-2) complete medium (Lonza). Cells were incubated at 37°C in a humidified atmosphere of 5% CO2 in the air.

### Transient transfection

As-T cells were cultured to 60% to 70% confluence in 35-mm dishes. The cells were transfected with 100 nM small interfering RNA (siRNA) oligos targeting human IL-8 (siIL-8) or scrambled control siRNA (siCtrl) using the jetPRIME reagent according to the manufacturer’s instruction.

### Establishment of IL-8 knockout (KO) stable cells

IL-8 was completely knocked out from As-T cells using the CRISPR/Cas9 technique. The lentivirus carrying Cas9 from Dharmacon (CAS10138, Lafayette, CO, USA) was packaged in 293T cells following the manufacturer’s instruction. The supernatants containing the virus were collected at 48, 60, and 72 h after transfection, and the viral soup was used to infect As-T cells in the presence of polybrene (10 μg/ml). After 72 h, blasticidin (1.5 μg/ml) was used to select cells stably expressing Cas9. The lentivirus carrying control sgRNA or IL-8 sgRNA (Abm, Richmond, Canada) was packaged in 293T cells as described above. The Cas9-expressing As-T cells were transduced with a viral soup containing the control or IL-8 sgRNA and then selected with G418 (100 μg/ml). The KO of IL-8 was confirmed using an immunoblotting assay.

### Vascular endothelial cells stably expressing eGFP

Lentivirus harboring an eGFP coding cassette (Transomics, Huntsville, AL, USA) was packaged in 293T cells as described above. HUVECs or HMVECs were transduced by lentiviral soup and selected by puromycin (1.0 μg/ml) to establish stable cells expressing the GFP protein.

### Isolation of total RNAs, RT-PCR, and qRT-PCR analysis

Total RNAs were extracted from cultured cells using TRIzol reagent (Invitrogen, Carlsbad, CA, USA). To determine the mRNA expression of IL-8, 1.0 μg total RNA was reverse transcribed to cDNA using the iScript cDNA synthesis kit (Bio-Rad, CA, USA) according to the manufacturer’s instruction. The IL-8 mRNA level was measured using the SYBR green supermix kit (Bio-Rad, CA, USA) according to the manufacturer’s instruction. GAPDH was used as an internal control. The primers were as follows: IL-8 forward primer, 5′-GAGAGTGATTGAGAGTGGACCAC-3′; IL-8 reverse primer, 5′-CACAACCCTCTGCACCCAGTTT-3′; GAPDH forward primer, 5′-TGTTGCCATCA ATGACCCCTT-3′; GAPDH reverse primer, 5′-CTCCACGACGTACTCAGCG-3′. The fold changes were calculated with the 2^–ΔΔCt^ method.

### ELISA assay

ELISA assay for determining IL-8 concentration in the conditioned medium of cells was performed using a human IL-8 ELISA kit according to the manufacturer’s instruction (D8000C, R&D Systems, Minneapolis, MN, USA). Optical densities were read at 405 nm on a microplate reader.

### Immunoblotting assay

Total cellular protein extracts were prepared using the RIPA buffer supplemented with protease and phosphatase inhibitors. Protein concentrations were determined using a BCA assay (Thermo Scientific, Philadelphia, PA, USA). Aliquots of 20 μg protein lysates were fractionated by 10% sodium dodecyl sulfate-polyacrylamide gel electrophoresis and transferred to a nitrocellulose membrane (Roche, Switzerland). Membranes were blocked with 5% non-fat dry milk for 2 h and then incubated with the following primary antibodies: IL-8 (1:1000, Cell Signaling), CXCR1 (1:1000, Abcam), CXCR2 (1:1000, Abcam), mTOR (1:1000, Cell Signaling), p-mTOR (1:1000, Cell Signaling), STAT3 (1:1000, Cell Signaling), p-STAT3 (1:1000, Cell Signaling), ERK (1:1000, Cell Signaling), p-ERK (1:1000, Cell Signaling), and β-actin (1:1000, Santa Cruz) overnight at 4°C. The membranes were then incubated with anti-rabbit or anti-mouse secondary antibodies (1:1000, Invitrogen) for 1 h at room temperature after three washes with PBST. The protein bands were visualized with enhanced chemiluminescence reagent (Thermo Scientific, Rockford, IL, USA), and images were acquired with a ChemiDoc Imaging System (Bio-Rad, CA, USA). Quantification of the immunoblotting results was performed using the Image Lab software (Bio-Rad, CA, USA).

### Wound healing assay

HUVECs or HMVECs alone or mixed with B2B or As-T cells at a 1:1 ratio were cultured until they reached almost 100% confluence in 6-well plates. The cell monolayer was scratched using a 200 μL pipette tip. The cells were washed twice with PBS to remove debris, and the wound was imaged at 0 h and 20 h using a Nikon ECLIPSE Ts2R microscope (Nikon, Japan). The average percentage of wound healing was determined based on measurements of three sites of the wound area.

### Transwell migration assay

Transwell chambers (from BD Biosciences, Bedford, MA and Costar, Corning Inc., Corning, NY, USA) with 8-μm pores on the membrane were inserted into 24-well plates. About 3.0 × 10^4^ HUVECs or HMVECs in 200 μL basic EBM2 medium (without growth factors and serum) were seeded onto each upper well of transwell chambers. The lower chamber of the transwell device was filled with 600 μL conditioned medium. After incubation for 24 h at 37°C, cells that did not migrate through the membrane were removed from the top well using a cotton swab. Cells that had migrated through the membrane were fixed with 20% methanol and stained with 0.1% crystal violet (Sigma, Saint Louis, MO, USA), imaged, and counted. The images were acquired with a Nikon ECLIPSE Ts2R microscope (Nikon, Japan). Three independent experiments were conducted in triplicate.

### Tube formation assay

Tube formation assay (angiogenesis assay *in vitro*) was performed as we previously described [16]. In brief, HUVECs were cultured in EBM-2 basal medium containing 0.2% FBS for 24 h before performing the tube formation assay. The conditioned media were prepared from different 100% confluent cells using EBM-2 medium with 1% FBS after culture for 24 h. The HUVECs were resuspended in EBM-2 basic medium, mixed with an equal volume of the conditioned medium, and seeded on Matrigel-pretreated 96-well plates at 2 × 10^4^ cells/well. Tube formation was observed and photographed after being cultured for indicated time points under the Nikon ECLIPSE Ci microscope (Nikon, Japan). The number and lengths of the tubular structures (the perimeter of each tubular structure) in each well were measured using the Angiogenesis Analyzer plugin of Image J (NIH, USA).

### Chicken chorioallantoic membrane (CAM) assay for tumor-induced angiogenesis

The CAM assay is performed as previously described [48]. White Leghorn fertilized chicken eggs were incubated at 37°C under constant humidity. An artificial air sac was created over a region containing small blood vessels in the CAM. The cells were implanted onto the CAM. Tumor angiogenesis was analyzed 4 days after the implantation. The area around the implanted Matrigel was photographed, and the blood vessel branches on the CAM were counted by two observers in a double-blind manner. Ten chicken embryos were used for each treatment group.

### Chimeric tumor model to test tumor growth using chicken CAM

Using chimeric tumor model to test tumor growth on CAM was performed as previously described [49]. Briefly, poly-l-lactide glycolide acid (PLGA) was used to make a biodegradable polymer matrix as we described [49]. White Leghorn fertilized chicken eggs were incubated at 37°C under constant humidity, and a small window was cut in the shell over the artificial air sac. The HMVECs (0.9 × 10^6^) were mixed with 0.1 × 10^6^ As-T cells and then mixed with 1:1 (v/v) growth factor-reduced Matrigel (BD Labware, USA). The As-T cells alone (0.1 × 10^6^) mixed with Matrigel were used as a control. The cell mixture was seeded into the PLGA sponges, incubated at 37°C for 30 min, and transplanted onto CAM. The eggshell window was resealed, and the chicken embryos were incubated at 37°C for tumor growth. After 12 days, the tumors were trimmed out from the CAM, photographed, and weighed.

### HMVEC/As-T co-implantation animal model

Ten BALB/c nude mice (5-week-old) were used for animal studies. The HMVECs (0.9 × 10^6^) were mixed with 0.1 × 10^6^ wild type control or IL-8-null As-T cells and then mixed with growth factor reduced Matrigel (BD, USA) in 1:1 (v/v) ratio. The mixtures were injected subcutaneously into both sides of the posterior flank of five nude mice in each group. Forty-five days later, all mice were sacrificed, and tumors were harvested. The long and short dimensions of the tumors were measured, and tumor sizes were calculated by the formula: volume (mm^3^) = [width^2^ (mm^2^) × length (mm)]/2. Part of the tumors was snap-frozen and stored at −80 degrees for later use; part of the tumors was formalin-fixed, paraffin-embedded, and sectioned at 5 μm for CD31 immunohistochemical staining under the standard procedure. The images were acquired with the Nikon ECLIPSE Ci microscope (Nikon, Japan). The CD31-positive microvessels were quantified. All procedures were approved by the Animal Care and Use Committee of Thomas Jefferson University.

### Statistical analysis

All statistical analysis was performed using GraphPad Prism 8 software (GraphPad Software, Inc., CA, USA). All the results are expressed as mean ± standard error (SE) of at least three individual experiments or each group. The means of two independent groups were compared using the unpaired Student’s *t*-test. The means of more than two groups were analyzed using the one-way *ANOVA* test followed by the *post hoc* Bonferroni test. The growth curves were analyzed using the two-way *ANOVA* test followed by the *post hoc* Bonferroni or Dunn test. A *P*-value less than 0.05 was considered statistically significant.

## Supplementary Materials

Supplementary Figures

## References

[1] Baker BA, Cassano VA, Murray C, and ACOEM Task Force on Arsenic Exposure. Arsenic Exposure, Assessment, Toxicity, Diagnosis, and Management: Guidance for Occupational and Environmental Physicians. J Occup Environ Med. 2018; 60:e634–9. 10.1097/JOM.0000000000001485 30358658

[2] Chung JY, Yu SD, Hong YS. Environmental source of arsenic exposure. J Prev Med Public Health. 2014; 47:253–7. 10.3961/jpmph.14.036 25284196 PMC4186553

[3] Palma-Lara I, Martínez-Castillo M, Quintana-Pérez JC, Arellano-Mendoza MG, Tamay-Cach F, Valenzuela-Limón OL, García-Montalvo EA, Hernández-Zavala A. Arsenic exposure: A public health problem leading to several cancers. Regul Toxicol Pharmacol. 2020; 110:104539. 10.1016/j.yrtph.2019.104539 31765675

[4] Nurchi VM, Djordjevic AB, Crisponi G, Alexander J, Bjørklund G, Aaseth J. Arsenic Toxicity: Molecular Targets and Therapeutic Agents. Biomolecules. 2020; 10:235. 10.3390/biom10020235 32033229 PMC7072575

[5] Putila JJ, Guo NL. Association of arsenic exposure with lung cancer incidence rates in the United States. PLoS One. 2011; 6:e25886. 10.1371/journal.pone.0025886 22003413 PMC3189216

[6] Tapio S, Grosche B. Arsenic in the aetiology of cancer. Mutat Res. 2006; 612:215–46. 10.1016/j.mrrev.2006.02.001 16574468

[7] Zhou Q, Xi S. A review on arsenic carcinogenesis: Epidemiology, metabolism, genotoxicity and epigenetic changes. Regul Toxicol Pharmacol. 2018; 99:78–88. 10.1016/j.yrtph.2018.09.010 30223072

[8] Hubaux R, Becker-Santos DD, Enfield KS, Rowbotham D, Lam S, Lam WL, Martinez VD. Molecular features in arsenic-induced lung tumors. Mol Cancer. 2013; 12:20. 10.1186/1476-4598-12-20 23510327 PMC3626870

[9] Gimbrone MA Jr, Leapman SB, Cotran RS, Folkman J. Tumor angiogenesis: iris neovascularization at a distance from experimental intraocular tumors. J Natl Cancer Inst. 1973; 50:219–28. 10.1093/jnci/50.1.219 4692862

[10] Semenza GL. Angiogenesis in ischemic and neoplastic disorders. Annu Rev Med. 2003; 54:17–28. 10.1146/annurev.med.54.101601.152418 12359828

[11] Liu LZ, Jiang Y, Carpenter RL, Jing Y, Peiper SC, Jiang BH. Role and mechanism of arsenic in regulating angiogenesis. PLoS One. 2011; 6:e20858. 10.1371/journal.pone.0020858 21687637 PMC3110823

[12] Zhou Q, Jin P, Liu J, Li S, Liu W, Xi S. Arsenic-induced HER2 promotes proliferation, migration and angiogenesis of bladder epithelial cells via activation of multiple signaling pathways in vitro and in vivo. Sci Total Environ. 2021; 753:141962. 10.1016/j.scitotenv.2020.141962 32890875

[13] Zhang J, Zhang Y, Wang W, Zhang Z. Potential molecular mechanisms underlying the effect of arsenic on angiogenesis. Arch Pharm Res. 2019; 42:962–76. 10.1007/s12272-019-01190-5 31701373

[14] Wang Z, Humphries B, Xiao H, Jiang Y, Yang C. Epithelial to mesenchymal transition in arsenic-transformed cells promotes angiogenesis through activating β-catenin-vascular endothelial growth factor pathway. Toxicol Appl Pharmacol. 2013; 271:20–9. 10.1016/j.taap.2013.04.018 23643801 PMC3714366

[15] Mittal V, El Rayes T, Narula N, McGraw TE, Altorki NK, Barcellos-Hoff MH. The Microenvironment of Lung Cancer and Therapeutic Implications. Adv Exp Med Biol. 2016; 890:75–110. 10.1007/978-3-319-24932-2_5 26703800

[16] Hisano Y, Hla T. Bioactive lysolipids in cancer and angiogenesis. Pharmacol Ther. 2019; 193:91–8. 10.1016/j.pharmthera.2018.07.006 30048709 PMC6309747

[17] Altorki NK, Markowitz GJ, Gao D, Port JL, Saxena A, Stiles B, McGraw T, Mittal V. The lung microenvironment: an important regulator of tumour growth and metastasis. Nat Rev Cancer. 2019; 19:9–31. 10.1038/s41568-018-0081-9 30532012 PMC6749995

[18] Wang X, Shen Y, Li S, Lv M, Zhang X, Yang J, Wang F, Yang J. Importance of the interaction between immune cells and tumor vasculature mediated by thalidomide in cancer treatment (Review). Int J Mol Med. 2016; 38:1021–9. 10.3892/ijmm.2016.2724 27599781

[19] Waugh DJ, Wilson C. The interleukin-8 pathway in cancer. Clin Cancer Res. 2008; 14:6735–41. 10.1158/1078-0432.CCR-07-4843 18980965

[20] Singh RD, Tiwari R, Khan H, Kumar A, Srivastava V. Arsenic exposure causes epigenetic dysregulation of IL-8 expression leading to proneoplastic changes in kidney cells. Toxicol Lett. 2015; 237:1–10. 10.1016/j.toxlet.2015.05.014 26008221

[21] Liu S, Sun Q, Wang F, Zhang L, Song Y, Xi S, Sun G. Arsenic induced overexpression of inflammatory cytokines based on the human urothelial cell model in vitro and urinary secretion of individuals chronically exposed to arsenic. Chem Res Toxicol. 2014; 27:1934–42. 10.1021/tx5002783 25257954

[22] Xu Y, Zhao Y, Xu W, Luo F, Wang B, Li Y, Pang Y, Liu Q. Involvement of HIF-2α-mediated inflammation in arsenite-induced transformation of human bronchial epithelial cells. Toxicol Appl Pharmacol. 2013; 272:542–50. 10.1016/j.taap.2013.06.017 23811328

[23] Yuan A, Chen JJ, Yao PL, Yang PC. The role of interleukin-8 in cancer cells and microenvironment interaction. Front Biosci. 2005; 10:853–65. 10.2741/1579 15569594

[24] Li A, Dubey S, Varney ML, Dave BJ, Singh RK. IL-8 directly enhanced endothelial cell survival, proliferation, and matrix metalloproteinases production and regulated angiogenesis. J Immunol. 2003; 170:3369–76. 10.4049/jimmunol.170.6.3369 12626597

[25] Lugano R, Ramachandran M, Dimberg A. Tumor angiogenesis: causes, consequences, challenges and opportunities. Cell Mol Life Sci. 2020; 77:1745–70. 10.1007/s00018-019-03351-7 31690961 PMC7190605

[26] Feng T, Yu H, Xia Q, Ma Y, Yin H, Shen Y, Liu X. Cross-talk mechanism between endothelial cells and hepatocellular carcinoma cells via growth factors and integrin pathway promotes tumor angiogenesis and cell migration. Oncotarget. 2017; 8:69577–93. 10.18632/oncotarget.18632 29050226 PMC5642501

[27] Chen QY, Li J, Sun H, Wu F, Zhu Y, Kluz T, Jordan A, DesMarais T, Zhang X, Murphy A, Costa M. Role of miR-31 and SATB2 in arsenic-induced malignant BEAS-2B cell transformation. Mol Carcinog. 2018; 57:968–77. 10.1002/mc.22817 29603397 PMC6588163

[28] Li L, Bi Z, Wadgaonkar P, Lu Y, Zhang Q, Fu Y, Thakur C, Wang L, Chen F. Metabolic and epigenetic reprogramming in the arsenic-induced cancer stem cells. Semin Cancer Biol. 2019; 57:10–18. 10.1016/j.semcancer.2019.04.003 31009762 PMC6690805

[29] He J, Wang M, Jiang Y, Chen Q, Xu S, Xu Q, Jiang BH, Liu LZ. Chronic arsenic exposure and angiogenesis in human bronchial epithelial cells via the ROS/miR-199a-5p/HIF-1α/COX-2 pathway. Environ Health Perspect. 2014; 122:255–61. 10.1289/ehp.1307545 24413338 PMC3948041

[30] Tsai CH, Yang MH, Hung AC, Wu SC, Chiu WC, Hou MF, Tyan YC, Wang YM, Yuan SF. Identification of Id1 as a downstream effector for arsenic-promoted angiogenesis via PI3K/Akt, NF-κB and NOS signaling. Toxicol Res (Camb). 2015; 5:151–9. 10.1039/c5tx00280j 30090333 PMC6062373

[31] Meng D, Wang X, Chang Q, Hitron A, Zhang Z, Xu M, Chen G, Luo J, Jiang B, Fang J, Shi X. Arsenic promotes angiogenesis in vitro via a heme oxygenase-1-dependent mechanism. Toxicol Appl Pharmacol. 2010; 244:291–9. 10.1016/j.taap.2010.01.004 20083128

[32] Soucy NV, Ihnat MA, Kamat CD, Hess L, Post MJ, Klei LR, Clark C, Barchowsky A. Arsenic stimulates angiogenesis and tumorigenesis in vivo. Toxicol Sci. 2003; 76:271–9. 10.1093/toxsci/kfg231 12970581

[33] Rajabi M, Mousa SA. The Role of Angiogenesis in Cancer Treatment. Biomedicines. 2017; 5:34. 10.3390/biomedicines5020034 28635679 PMC5489820

[34] Nishida N, Yano H, Nishida T, Kamura T, Kojiro M. Angiogenesis in cancer. Vasc Health Risk Manag. 2006; 2:213–9. 10.2147/vhrm.2006.2.3.213 17326328 PMC1993983

[35] Xie K. Interleukin-8 and human cancer biology. Cytokine Growth Factor Rev. 2001; 12:375–91. 10.1016/s1359-6101(01)00016-8 11544106

[36] Sharma I, Singh A, Siraj F, Saxena S. IL-8/CXCR1/2 signalling promotes tumor cell proliferation, invasion and vascular mimicry in glioblastoma. J Biomed Sci. 2018; 25:62. 10.1186/s12929-018-0464-y 30086759 PMC6081798

[37] Bar-Eli M. Role of interleukin-8 in tumor growth and metastasis of human melanoma. Pathobiology. 1999; 67:12–8. 10.1159/000028045 9873223

[38] Gabellini C, Trisciuoglio D, Desideri M, Candiloro A, Ragazzoni Y, Orlandi A, Zupi G, Del Bufalo D. Functional activity of CXCL8 receptors, CXCR1 and CXCR2, on human malignant melanoma progression. Eur J Cancer. 2009; 45:2618–27. 10.1016/j.ejca.2009.07.007 19683430

[39] Long X, Ye Y, Zhang L, Liu P, Yu W, Wei F, Ren X, Yu J. IL-8, a novel messenger to cross-link inflammation and tumor EMT via autocrine and paracrine pathways (Review). Int J Oncol. 2016; 48:5–12. 10.3892/ijo.2015.3234 26548401

[40] Li A, Varney ML, Valasek J, Godfrey M, Dave BJ, Singh RK. Autocrine role of interleukin-8 in induction of endothelial cell proliferation, survival, migration and MMP-2 production and angiogenesis. Angiogenesis. 2005; 8:63–71. 10.1007/s10456-005-5208-4 16132619

[41] Sparmann A, Bar-Sagi D. Ras-induced interleukin-8 expression plays a critical role in tumor growth and angiogenesis. Cancer Cell. 2004; 6:447–58. 10.1016/j.ccr.2004.09.028 15542429

[42] Holmes WE, Lee J, Kuang WJ, Rice GC, Wood WI. Structure and functional expression of a human interleukin-8 receptor. Science. 1991; 253:1278–80. 10.1126/science.1840701 1840701

[43] Venkatakrishnan G, Salgia R, Groopman JE. Chemokine receptors CXCR-1/2 activate mitogen-activated protein kinase via the epidermal growth factor receptor in ovarian cancer cells. J Biol Chem. 2000; 275:6868–75. 10.1074/jbc.275.10.6868 10702246

[44] Luppi F, Longo AM, de Boer WI, Rabe KF, Hiemstra PS. Interleukin-8 stimulates cell proliferation in non-small cell lung cancer through epidermal growth factor receptor transactivation. Lung Cancer. 2007; 56:25–33. 10.1016/j.lungcan.2006.11.014 17175059

[45] Wu J, Gao FX, Wang C, Qin M, Han F, Xu T, Hu Z, Long Y, He XM, Deng X, Ren DL, Dai TY. IL-6 and IL-8 secreted by tumour cells impair the function of NK cells via the STAT3 pathway in oesophageal squamous cell carcinoma. J Exp Clin Cancer Res. 2019; 38:321. 10.1186/s13046-019-1310-0 31324197 PMC6642486

[46] Guo Y, Zang Y, Lv L, Cai F, Qian T, Zhang G, Feng Q. IL-8 promotes proliferation and inhibition of apoptosis via STAT3/AKT/NF-κB pathway in prostate cancer. Mol Med Rep. 2017; 16:9035–42. 10.3892/mmr.2017.7747 29039490

[47] MacManus CF, Pettigrew J, Seaton A, Wilson C, Maxwell PJ, Berlingeri S, Purcell C, McGurk M, Johnston PG, Waugh DJ. Interleukin-8 signaling promotes translational regulation of cyclin D in androgen-independent prostate cancer cells. Mol Cancer Res. 2007; 5:737–48. 10.1158/1541-7786.MCR-07-0032 17606477

[48] Xu Q, Liu LZ, Yin Y, He J, Li Q, Qian X, You Y, Lu Z, Peiper SC, Shu Y, Jiang BH. Regulatory circuit of PKM2/NF-κB/miR-148a/152-modulated tumor angiogenesis and cancer progression. Oncogene. 2015; 34:5482–93. 10.1038/onc.2015.6 25703326

[49] Liu LZ, Zheng JZ, Wang XR, Jiang BH. Endothelial p70 S6 kinase 1 in regulating tumor angiogenesis. Cancer Res. 2008; 68:8183–8. 10.1158/0008-5472.CAN-08-0819 18829578

