# Supplementary material for: Human endothelial cells promote arsenic-transformed lung epithelial cells to induce tumor growth and angiogenesis through interleukin-8 induction
Source: Aging (Albany NY). 2022 Mar 3;14(5):2113–30. doi: 10.18632/aging.203930 (PMC8954972; doi:10.18632/aging.203930)
Supplement: Supplementary Figures [file aging-14-203930-s001.pdf]

SUPPLEMENTARY FIGURES

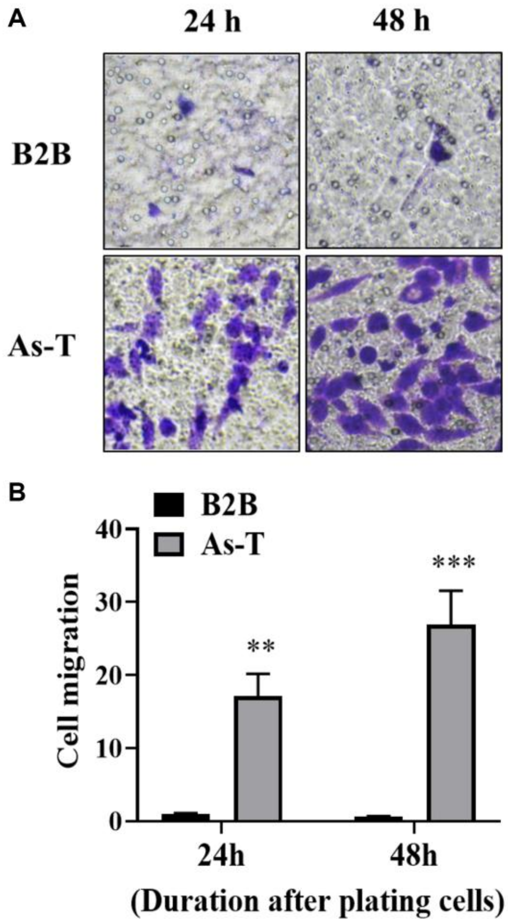

Supplementary Figure 1. Medium from As-T cells promoted the transwell migratory ability of HUVEC cells. B2B and As-T cells were plated on the lower chamber of 24 well-plate. When the cells were confluent, 600 μL 1% FBS supplemented DMEM medium was added to the wells and incubated for 24 h. The starved HUVEC cells were resuspended in the basic EBM2 medium and plated in the top chamber of the transwell cassette. The migrated cells were stained and counted at 24 and 48 h. Images were acquired at 10× magnification. (**A**) Representative images of migrated cells. (**B**) Quantification of the migrated cells. ** and $^{***}p < 0.01$ and $p < 0.001$, respectively, compared to CM from B2B cells.

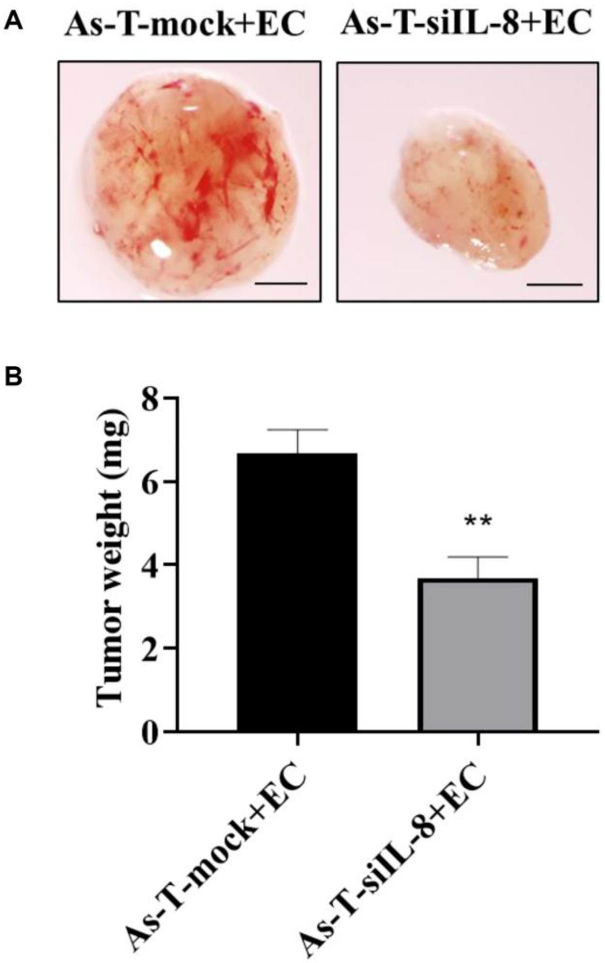

Supplementary Figure 2. Silencing IL-8 in As-T cells suppressed the promoting effect of endothelial cells on As-T cell-induced tumor growth. Mock control or siIL-8-transfected As-T cells were mixed with HMVECs in a 1:9 ratio in serum-free medium, and then the mixed cells were carried by the PLGA sponges for the implantation onto the CAM of 8 days old chicken embryos. The tumors were harvested 12 days after the implantation. (**A**) Representative tumors. Scale, 1 mm. (**B**) The weight of plugs (n = 6 for each group). $^{**}p < 0.01$ compared with mock As-T+EC group.
